# Analysis of the genetic variation in mitochondrial DNA, Y-chromosome sequences, and *MC1R* sheds light on the ancestry of Nigerian indigenous pigs

**DOI:** 10.1186/s12711-017-0326-1

**Published:** 2017-06-26

**Authors:** Adeniyi C. Adeola, Olufunke O. Oluwole, Bukola M. Oladele, Temilola O. Olorungbounmi, Bamidele Boladuro, Sunday C. Olaogun, Lotanna M. Nneji, Oscar J. Sanke, Philip M. Dawuda, Ofelia G. Omitogun, Laurent Frantz, Robert W. Murphy, Hai-Bing Xie, Min-Sheng Peng, Ya-Ping Zhang

**Affiliations:** 1 State Key Laboratory of Genetic Resources and Evolution, Yunnan Laboratory of Molecular Biology of Domestic Animals, Kunming Institute of Zoology, Chinese Academy of Sciences, Kunming, China; 2 Sino-Africa Joint Research Center, Chinese Academy of Sciences, Kunming, China; 3 Institute of Agricultural Research and Training, Obafemi Awolowo University, Ibadan, Nigeria; 4 Department of Veterinary Medicine, University of Ibadan, Ibadan, Nigeria; 5 Taraba State Ministry of Agriculture and Natural Resources, Jalingo, Nigeria; 6 Department of Veterinary Surgery and Theriogenology, College of Veterinary Medicine, University of Agriculture Makurdi, Makurdi, Nigeria; 7 Department of Animal Sciences, Obafemi Awolowo University, Ile-Ife, Nigeria; 8 The Palaeogenomics and Bio-Archaeology Research Network, Research Laboratory for Archaeology, University of Oxford, Oxford, UK; 9 School of Biological and Chemical Sciences, Queen Mary University of London, London, UK; 10 Centre for Biodiversity and Conservation Biology, Royal Ontario Museum, Toronto, Canada; 11 Kunming College of Life Science, University of Chinese Academy of Sciences, Kunming, China; 12 State Key Laboratory for Conservation and Utilization of Bio-Resources in Yunnan, Yunnan University, Kunming, China

## Abstract

**Background:**

The history of pig populations in Africa remains controversial due to insufficient evidence from archaeological and genetic data. Previously, a Western ancestry for West African pigs was reported based on loci that are involved in the determination of coat color. We investigated the genetic diversity of Nigerian indigenous pigs (NIP) by simultaneously analyzing variation in mitochondrial DNA (mtDNA), Y-chromosome sequence and the *melanocortin receptor 1* (*MC1R*) gene.

**Results:**

Median-joining network analysis of mtDNA D-loop sequences from 201 NIP and previously characterized loci clustered NIP with populations from the West (Europe/North Africa) and East/Southeast Asia. Analysis of partial sequences of the Y-chromosome in 57 Nigerian boars clustered NIP into lineage HY1. Finally, analysis of *MC1R* in 90 NIP resulted in seven haplotypes, among which the European wild boar haplotype was carried by one individual and the European dominant black by most of the other individuals (93%). The five remaining unique haplotypes differed by a single synonymous substitution from European wild type, European dominant black and Asian dominant black haplotypes.

**Conclusions:**

Our results demonstrate a European and East/Southeast Asian ancestry for NIP. Analyses of *MC1R* provide further evidence. Additional genetic analyses and archaeological studies may provide further insights into the history of African pig breeds. Our findings provide a valuable resource for future studies on whole-genome analyses of African pigs.

**Electronic supplementary material:**

The online version of this article (doi:10.1186/s12711-017-0326-1) contains supplementary material, which is available to authorized users.

## Background

The origins of African pig breeds are highly controversial owing to a paucity of archaeological and genetic data for hypothesis testing [1, 2]. Previous genetic analyses of West African pigs revealed that they shared maternal and paternal haplotypes with European wild boars and pigs, but not with Near Eastern wild boars [3]. The limited size of West African pig samples did not allow discriminating them from pigs domesticated in North Africa and/or from pigs introduced by the European colonizers during the 15th–19th centuries. Early Portuguese sailors circumnavigated Africa and, in doing so, they may have introduced a European gene pool into some West African pigs [1]. However, this hypothesis has not been formally tested through genetic analysis. Some Iberian pigs are classified as black hairy and this pattern is common in indigenous West African pigs [1]. However, the causal genetic variants that underlie the color phenotype in the latter pigs remain largely unexplored. Melanocortin receptor 1 (*MC1R*) is a major determinant in color phenotype [4]. Functional mutations in *MC1R* result in different coat colors in domestic animals, such as cattle [5], horses [6], goats [7], sheep [8–10] and pigs [11–13]. Research on *MC1R* has provided valuable insights into the evolution of domesticated animals [13–15]. For instance, Linderholm et al. [13] showed that, among the alleles of *MC1R*, there is a novel black allele unique to Polynesian pigs. Therefore, we used this gene to investigate the genetic diversity and origin of hairy black Nigerian indigenous pigs (NIP) as well as data from mitochondrial DNA (mtDNA) and Y-chromosomes of NIP to provide insights into the origin of NIP.

## Methods

### Animals

Peripheral blood samples were collected from 204 NIP distributed in six Nigeria states after receiving appropriate permission from their owners (see Additional file 1: Table S1).

### Analysis of mtDNA D-loop sequences

Our data involved the amplification and sequencing of 630-base pair (bp) fragments of mtDNA D-loop (the methods are detailed in Additional file 2; GenBank accession numbers: KU561971–KU562068 and KY055561–KY055663). The final dataset for analysis comprised 201 NIP (de novo) and 722 mtDNA D-loop sequences of pigs retrieved from GenBank (see Additional file 3: Table S2). All 923 sequences were aligned and trimmed to 464 bp, which corresponded to nucleotide positions between 112 and 575 of the reference sequence EF545567 [16]. A median-joining network of 923 pig sequences was constructed using NETWORK 5.0 [17].

### Y-chromosome analysis

Paternal genetic data were also obtained from 57 Nigerian indigenous sires (see Additional file 1: Table S1) by sequencing 370 bp of intron 1 and part of the flanking exons 1 and 2 of the Y-linked gene *UTY* (*ubiquitously transcribed tetratricopeptide repeat*), which contains repeats (see methods in Additional file 2; GenBank accession numbers: KU561941–KU561970 and KY234314–KY234340). Single nucleotide polymorphisms (SNPs) in the *UTY* amplicon were used to diagnose Y-chromosome lineages HY1 and HY2 versus HY3 [3].

### Analysis of *MC1R* sequences

Finally, we analyzed sequence variation in *MC1R* for 90 NIP (see Additional file 1: Table S1) by sequencing the entire *MC1R*-coding region i.e. 963 bp (see methods in Additional file 2; GenBank accession numbers: KX264504**–**KX264593).

## Results and discussion

### Mitochondrial DNA

**Fig. 1:**
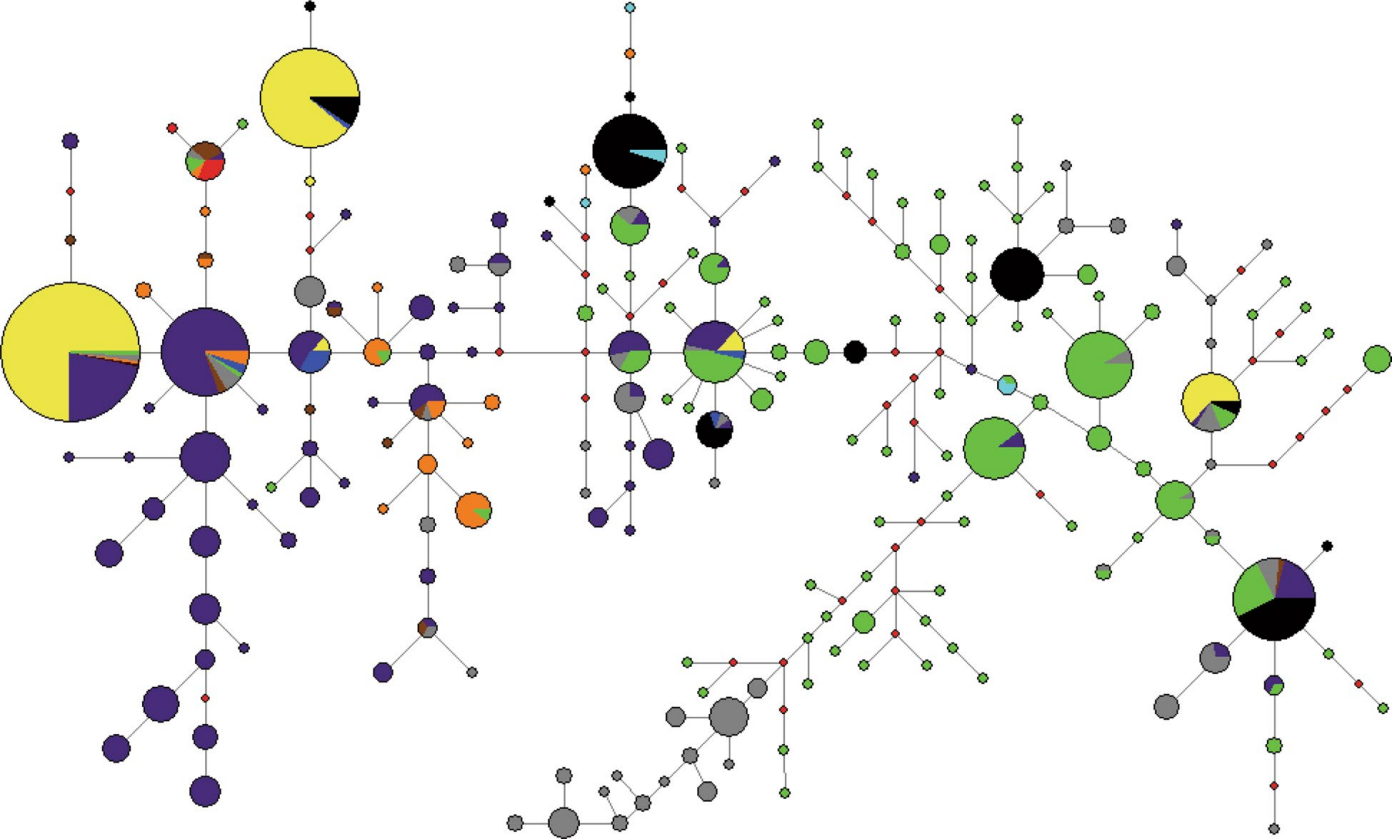
Median-joining network of 923 D-loop sequences corresponding to Nigerian indigenous pigs, the global population of pig and the wild boar population. NIP cluster with European and East/Southeast Asian pigs. *Colors* indicate locations: *yellow* indigenous pigs from Nigeria; *blue* United Kingdom; *orange* America; *brown* Iberia (Portugal + Spain); *black* East Africa (Uganda + Kenya + Zimbabwe); *grey* Indonesian pigs; *red* North Africa (Moroccan + Tunisian) wild boars; *lime* East Asian and mainland Southeast Asian (Japan, Korea, Vietnam, Thailand) pigs and wild boars; *purple* other European countries (Germany, Luxemburg, Belgium, Italy, Austria, France, Hungry); and *light blue* Indian pigs. *Note*: *red diamonds* denote intermediate haplotypes

NIP individuals clustered with pig individuals from both the West (Europe/North Africa) and East/Southeast Asia (Fig. 1). These results were consistent with previous analyses of West African pigs [3]. The early introduction of unimproved Iberian swine by the Portuguese into West Africa may have influenced NIP [1]. Ubiquitous standard European breeds, such as Large White and Landrace, which are white pigs, are widespread in Africa because of their excellent productivity, which often overcomes that of local populations [1]. Previously, genetic analyses of indigenous and commercially-developed crossbred pigs from southwestern Nigeria raised concerns about the possibility of genetic erosion in the locally-adapted pigs [18]. Introgression of the Asian matrilineal haplotype into European commercial pigs might have resulted in the clustering of some NIP with East/Southeast Asian pigs. It is also possible that the observed Asian haplotypes in NIP were inherited directly through female Asian introgression due to a low frequency of the European haplotype in NIP that carried the Asian haplotype (Fig. 1).

### Y-chromosome

All of the 57 analyzed Nigerian sires were assigned to the HY1 haplotype only (data not shown), which occurs widely in both Europe and Asia [3]. None of the NIP were assigned to HY3, which is unique to Asia and was detected at considerable high frequency in Kenyan pigs (35%) and Zimbabwean Mukota pigs (100%) [3]. This might be due to the influence of East/Southeast Asian pigs on African pigs and, particularly the Mukota pigs from Zimbabwe, which closely resemble the Chinese lard pig, in terms of morphology. Our finding agrees with that of an earlier study that reported Western ancestry for West African pigs [3].

### *MC1R* variation

**Fig. 2:**
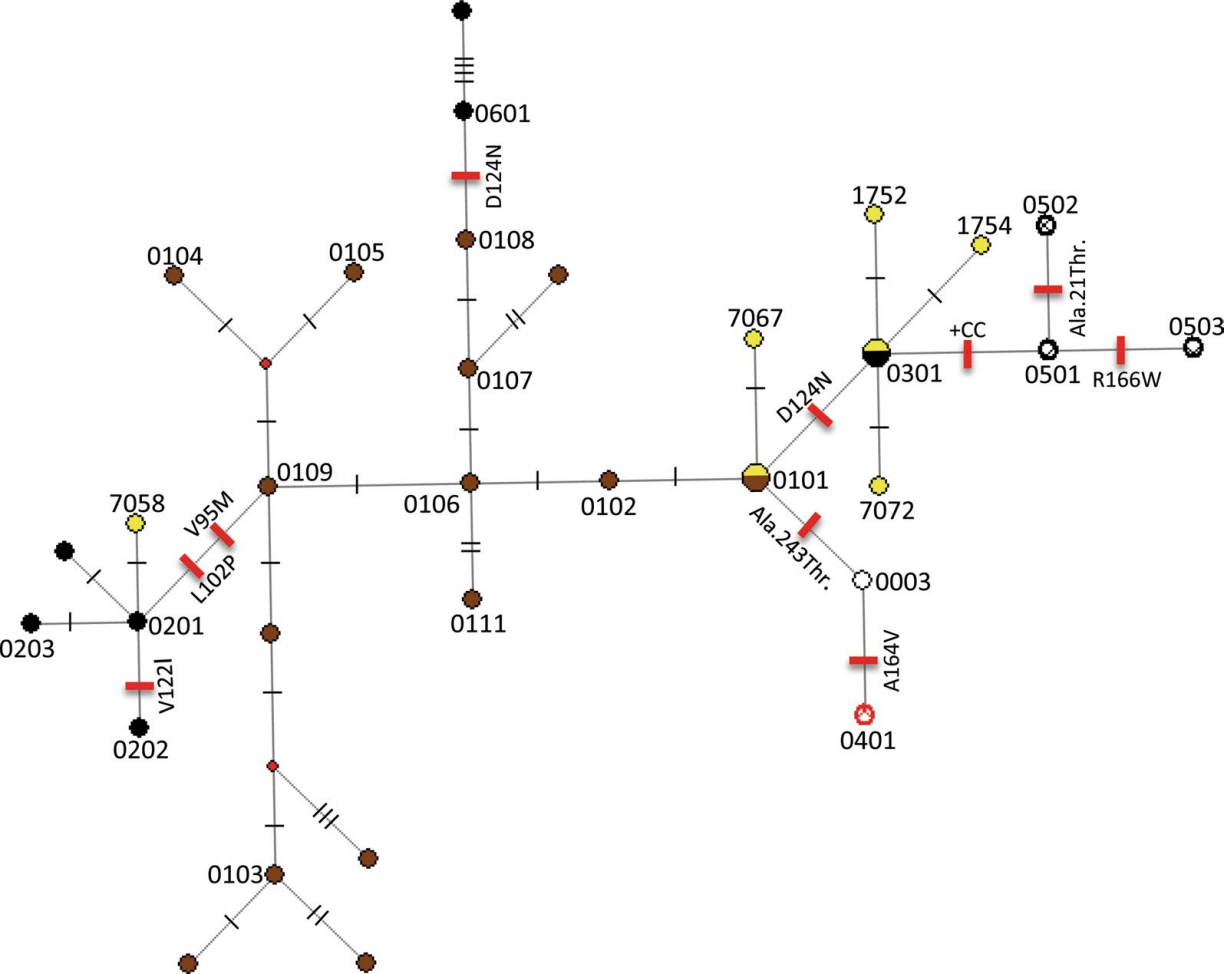
Median joining network of *MC1R* haplotypes in Nigerian, Polynesian, Asian and European pigs. All known haplotypes are represented by *circles*. *Colors* inside the *circles* indicate the type and nomenclature as follows [13, 14]: *brown* (E^+^ European and Asian—wild type); *yellow* Nigerian indigenous pigs (NIP); *red* (e—recessive red—European); *black* and *white* (E^P^—spotted black—European); and *black* (E^D2^ and E^D1^—dominant black—European, Polynesian and Asian). Differences in sequences are noted on each of the *branches* and *the small dash lines* represent the number of steps. *Red ticks* perpendicular to each branch represent non-synonymous mutations that change the protein sequence. *Note*: *red diamond symbols* represent intermediate haplotypes

Analyses using PHASE version 2.1.1 [19, 20] on the NIP samples led to the construction of seven haplotypes for *MC1R* (see Additional file 4: Table S3). The median joining network (Fig. 2) and Additional file 5: Table S4 show that there was one individual with the E^+^ European wild type *MC1R* haplotype [14]. Although this homozygous individual carried the European wild type, it displayed a variable coat color phenotype. Within the tested sample of NIP, the E^*D2*^ (European-dominant black) was the most frequent *MC1R* haplotype at 93% (see Additional file 3: Table S2). The remaining five unique haplotypes differed by a single synonymous substitution from the E^+^ (European wild type), E^*D2*^ (European dominant black) and Asian E^*D1*^ (dominant black) haplotypes (Fig. 2).

Direct selection for non-camouflage patterns was proposed to be an essential component of the selection of coat color loci in domestic animals [14], which may have been fostered by animal husbandry. Independent selective sweeps have been identified in Chinese and European pigs resulting in the dominant black color. For instance, in Polynesia and Europe, selection of pigs for the D124N substitution in *MC1R* resulted in a dominant black color, whereas selection for the L102P substitution in *MC1R* was responsible for the dominant black color in Chinese pigs [13–15]. These mutations have been used to differentiate Polynesian, European and Asian black pigs. Therefore, the high frequency of the European dominant black color haplotype in NIP suggests the occurrence of gene flow from local European pig breeds. The NIP individuals that carry the East Asian *MC1R* haplotype might have originated from European black pigs, which agrees with findings from a genome-wide analysis that showed that introgression of Asian haplotypes via anthropogenic hybridization and selection has influenced the genomic architecture of European pigs [21]. Similarly, another possibility is a direct Asian introgression in NIP. Future investigations based on evidence from whole-genome sequence data should test these possibilities (Additional file 2).

## Conclusions

In summary, this study reveals that NIP have mainly a European ancestry with some East/Southeast Asian ancestry, which may be due to direct introgression or through introgression from European pig breeds, themselves derived from introgression with Asian breeds. It also provides a first glimpse on *MC1R* variation across populations of indigenous pigs in one West Africa country. This study was designed to provide a valuable resource for future studies on whole-genome analyses of African pigs.

## Additional files

**Additional file 1: Table S1.** Data on the 204 hairy black NIP sampled from six states in Nigeria. Information on the mtDNA, Y-chromosome and *MC1R* of the 204 NIP samples.

**Additional file 2.** Details on blood sampling of NIP individuals and sequencing of mtDNA D-loop, Y-chromosome and *MC1R* sequences [15–17, 23]. This is comprehensive information on sampling and sequencing procedure for the 204 NIP.

**Additional file 3: Table S2.** Data on the 722 pig D-loop sequences analyzed in this study. European, African and Asian mitochondrial control region sequences retrieved from GenBank were used for the median joining network analysis (Fig. 1). Assignments to sub-haplogroups and variants conform to the pig and wild boar mtDNA tree obtained from DomeTree [22].

**Additional file 4: Table S3.** Mutations in the *MC1R* coding region defining seven haplotypes and their frequencies in Nigerian indigenous pigs. 0301 is the European dominant black pig haplotype; column NIP provides copy number of each haplotype among the samples; dots indicate identity with the previously reported [14] European wild boar haplotype (0101).

**Additional file 5: Table S4.** *MC1R* alleles in the global population of pigs. Thirty-four *MC1R* haplotypes (seven NIP *de novo* plus 27 downloaded from GenBank) were used to construct the *MC1R* network.

## References

[1] Blench RM, Blench RM, Mac Donald KC (2000). A history of pigs in Africa. The origins and development of African livestock: archaeology, genetics, linguistics and ethnography.

[2] Amills M, Ramırez O, Galman-Omitogun O, Clop A (2013). Domestic pigs in Africa. Afr Archaeol Rev.

[3] Ramirez O, Ojeda A, Tomas A, Gallardo D, Huang LS, Folch JM (2009). Integrating Y-chromosome, mitochondrial, and autosomal data to analyze the origin of pig breeds. Mol Biol Evol.

[4] Lin JY, Fisher DE (2007). Melanocyte biology and skin pigmentation. Nature.

[5] Rouzaud F, Martin J, Gallet PF, Delourme D, Goulemot-Leger V, Amigues Y (2000). A first genotyping assay of French cattle breeds based on a new haplotype of the extension gene encoding the melanocortin-1 receptor (*MC1R*). Genet Sel Evol.

[6] Marklund L, Moller MJ, Sandberg K, Andersson L (1996). A missense mutation in the gene for melanocyte-stimulating hormone receptor (MC1R) is associated with the chestnut coat color in horses. Mamm Genome.

[7] Fontanesi L, Beretti F, Riggio V, Dall’Olio S, Gonzalez EG, Finocchiaro R (2009). Missense and nonsense mutations in melanocortin 1 receptor (*MC1R*) gene of different goat breeds: association with red and black coat colour phenotypes but with unexpected evidences. BMC Genet.

[8] Vage DI, Klungland H, Lu D, Cone RD (1999). Molecular and pharmacological characterization of dominant black coat color in sheep. Mamm Genome.

[9] Vage DI, Fleet MR, Ponz R, Olsen RT, Monteagudo LV, Tejedor MT (2003). Mapping and characterization of the dominant black colour locus in sheep. Pigment Cell Res.

[10] Fontanesi L, Dall’Olio S, Beretti F, Portolano B, Russo V (2011). Coat colours in the Massese sheep breed are associated with mutations in the agouti signalling protein (*ASIP*) and melanocortin 1 receptor (*MC1R*) genes. Animal.

[11] Kijas JM, Wales R, Tornsten A, Chardon P, Moller M, Andersson L (1998). *Melanocortin receptor 1* (*MC1R*) mutations and coat color in pigs. Genetics.

[12] Kijas JM, Moller M, Plastow G, Andersson L (2001). A frameshift mutation in *MC1R* and a high frequency of somatic reversions cause black spotting in pigs. Genetics.

[13] Linderholm A, Spencer D, Battista V, Frantz L, Barnett R, Fleischer RC (2016). A novel *MC1R* allele for black coat colour reveals the Polynesian ancestry and hybridization patterns of Hawaiian feral pigs. R Soc Open Sci.

[14] Fang M, Larson G, Ribeiro HS, Li N, Andersson L (2009). Contrasting mode of evolution at a coat color locus in wild and domestic pigs. PLoS Genet.

[15] Li J, Yang H, Li JR, Li HP, Ning T, Pan XR (2010). Artificial selection of the melanocortin receptor 1 gene in Chinese domestic pigs during domestication. Heredity (Edinb).

[16] Wu GS, Yao YG, Qu KX, Ding ZL, Li H, Palanichamy MG (2007). Population phylogenomic analysis of mitochondrial DNA in wild boars and domestic pigs revealed multiple domestication events in East Asia. Genome Biol.

[17] Bandelt HJ, Forster P, Röhl A (1999). Median-joining networks for inferring intraspecific phylogenies. Mol Biol Evol.

[18] Adeola AC, Omitogun OG (2012). Characterization of indigenous pigs in Southwestern Nigeria using blood protein polymorphism. Anim Genet Resour.

[19] Stephens M, Smith NJ, Donnelly P (2001). A new statistical method for haplotype reconstruction from population data. Am J Hum Genet.

[20] Stephens M, Scheet P (2005). Accounting for decay of linkage disequilibrium in haplotype inference and missing-data imputation. Am J Hum Genet.

[21] Bosse M, Lopes MS, Madsen O, Megens HJ, Crooijmans RP, Frantz LA (2015). Artificial selection on introduced Asian haplotypes shaped the genetic architecture in European commercial pigs. Proc Biol Sci.

[22] Peng MS, Fan L, Shi NN, Ning T, Yao YG, Murphy RW (2015). DomeTree: a canonical toolkit for mitochondrial DNA analyses in domesticated animals. Mol Ecol Resour.

[23] Tamura K, Peterson D, Peterson N, Stecher G, Nei M, Kumar S (2011). MEGA5: molecular evolutionary genetics analysis using maximum likelihood, evolutionary distance, and maximum parsimony methods. Mol Biol Evol.

