# Supplementary material for: Analysis of the genetic variation in mitochondrial DNA, Y-chromosome sequences, and MC1R sheds light on the ancestry of Nigerian indigenous pigs
Source: Genet Sel Evol. 2017 Jun 26;49:52. doi: 10.1186/s12711-017-0326-1 (PMC5485568; doi:10.1186/s12711-017-0326-1)
Supplement: Supplementary file 2 — Additional file 2. Details on blood sampling of NIP individuals and sequencing of mtDNA D-loop, Y-chromosome and MC1R sequences [15–17, 23]. This is comprehensive information on sampling and sequencing procedure for the 204 NIP. [file 12711_2017_326_MOESM2_ESM.docx]

**Additional file 2: Appendix**

**Sampling**

The study involved drawing of ~5ml blood from 204 genetically unrelated hairy black NIP distributed in six different states that is more than 100 km apart in Nigeria (See Additional file 1: Table S1**).** Blood samples were kept in 95% ethanol at room temperature until taken to laboratory and stored at 4^o^C for immediate use, or -80^o^C for longer storage.

**mtDNA D-loop, Y-chromosome and *MC1R* PCR and sequencing methods**

1. Sequencing of mtDNA D-loop

Genomic DNA was extracted from whole blood by the standard phenol/chloroform method. The protocols of PCR amplification and sequencing of mtDNA D-loop were as follows: the 630 base pairs (bp) were amplified using primers (forward 5’-CAACCAAAACAAGCATTCCA-3’, reverse 5’-GATTGTGGGCGTATGCTTAAA-3’). The PCR amplification was conducted in a 15-ul final volume containing PCR buffer, 2.5 mM of MgCl2, 0.25 mM dNTPs, 0.3 um of each primer and 0.75 of U rTaq Gold DNA polymerase (Applied Biosystems). The thermocycling profile consisted of a cycle at 95^o^C for 10 min, followed by 35 cycles of 95^o^C for 1 min, 60^o^C for 1 min, 72^o^C for 1 min and a final extension step at 72^o^C for 7 min. The PCR products were purified using the ExoSAP-IT PCR Cleanup kit (Affymetrix) and sequencing was in both directions using the same sets of primers. Sequencing reactions were done with the Big Dye Terminator Cycle Sequencing Kit v1.1 (Applied Biosystems). The electropherograms for each of the sequences were visualized, edited and aligned by SeqMan Pro of DNASTAR Lasergene 7.1.0 (DNAStar Inc., Madison, WI) with the reference sequence EF545567 [16].

1. Sequencing of Y-Chromosome

Amplification was carried out with the primer (forward 5’-AGCTGTTTTCGGTGATGAGG-3’ and reverse 5’-TGCCCAACAGAGTTTTAGTCC-3’) in a 15-ul final volume containing PCR buffer, 2.5 mM of MgCl2, 0.25 mM dNTPs, 0.3 um of each primer and 0.75 of U rTaq Gold DNA polymerase (Applied Biosystems). The thermocycling profile consisted of a cycle at 95^o^C for 10 min, followed by 35 cycles of 95^o^C for 1 min, 60^o^C for 1 min, 72^o^C for 1 min and a final extension step at 72^o^C for 7 min. The PCR products were purified using the ExoSAP-IT PCR Cleanup kit (Affymetrix) and sequenced following the similar protocol employed for the mitochondrial D-loop region.

1. *MC1R* Sequencing

The MC1R fragment that included the entire coding region, 43 bp of 5’-UTR and 208 bp of 3’UTR from 90 individuals were amplified using the forward primer MF1 (5’-GTGCGGCGGCTCTGCGCTCCAA-3’) and the reverse primer (5’-CCCCCACTCCCCATGCCTCCTG-3’) [15]. The PCR profile included a 3 min initial denaturation at 94 ^o^C, followed by 35 cycles of: 1 min denaturation at 94^o^C, 1 min annealing at 64^o^C, 1 min of extension at 72^o^C and a final extension of 10 min at 72^o^C. The amplified products were purified with a gel extraction kit (Watson Biomedical Inc.) and sequenced following the similar protocol employed for the mitochondrial D-loop region using the primers listed below. All the sequences from each sample were aligned and edited by DNAStar Software (DNAStar Inc. Madison, WI, USA). Aligned sequence data were imported into MEGA 5.0 [23] for analyzing nucleotide composition and variable sites. A median-joining network [17] was also constructed using program NETWORK 4.2.01 (<http://www.fluxus-technology.com>) and modified manually to reveal evolutionary relationships among haplotypes.

**Sequencing primers**

| Sequencing primers | 5’ – 3’ | bp |
| --- | --- | --- |
| MC1RF1 | gTg Cgg Cgg CTC TgC gCT CCA A | 22 |
| MC1RR1 | CCC CCA CTC CCC ATg CCT CCT g | 22 |
| MC1RF2 | ggC ggC TCT gCg CTC CAA ggA | 21 |
| MC1RR2 | CgC CgT CTC TCC AgC CTC CCC | 21 |
| MC1RSF1 | gCg ggA CgA TgC CCg TgC TTg | 21 |
| MC1RSR1 | TgT ggT ggT AgT Agg CgA TgA | 21 |
| MC1RSF2 | Tgg Cgg gAC gAT gCC CgT gCT Tg | 23 |
| MC1RSF3 | CAC CCT CTT CAT CgC CTA CTA CC | 23 |
| MC1RSF4 | ggA ggA ggg gCT CAg gC | 17 |
| MC1RSF5 | CTT CAA gAA CgT CAA CC | 17 |
| MC1RSF6 | gTC TTC AAg AAC gTC AA | 17 |
| MC1RSR5 | CCA CCT CCC CAC CAA gT | 17 |
| MC1RSR6 | ggC TgC gCC CTT gAg gC | 17 |
| MC1RSF7 | ggg AAg ACT Tgg Tgg ggA | 18 |
| MC1RSR7 | ggg CCA AgC ACA ggC ATC gTC | 21 |
