# Supplementary material for: Analysis of the genetic variation in mitochondrial DNA, Y-chromosome sequences, and MC1R sheds light on the ancestry of Nigerian indigenous pigs
Source: Genet Sel Evol. 2017 Jun 26;49:52. doi: 10.1186/s12711-017-0326-1 (PMC5485568; doi:10.1186/s12711-017-0326-1)
Supplement: Supplementary file 4 — Additional file 4: Table S3. Mutations in the MC1R coding region defining seven haplotypes and their frequencies in Nigerian indigenous pigs. 0301 is the European dominant black pig haplotype; column NIP provides copy number of each haplotype among the samples; dots indicate identity with the previously reported [14] European wild boar haplotype (0101). [file 12711_2017_326_MOESM4_ESM.docx]

| Haplotype | 2 | 4 | 17 | 21 | 22 | 95 | 102 | 117 | 121 | 122 | 124 | 164 | 166 | 227 | 242 | 243 | 301 | NIP |
| --- | --- | --- | --- | --- | --- | --- | --- | --- | --- | --- | --- | --- | --- | --- | --- | --- | --- | --- |
| 0101 | T | T | G | C | C | G | T | G | T | C | G | G | G | C | C | G | C | 2 |
| 0301 | . | . | . | . | . | . | . | . | . | . | A | . | . | . | . | . | . | 168 |
| 1752 | . | . | . | . | . | . | . | . | . | . | A | . | . | A | . | . | . | 2 |
| 1754 | . | . | . | . | . | . | . | . | . | . | A | . | . | . | G | . | . | 2 |
| 7072 | C | . | . | . | . |  | . | . | . | . | A | . | . | . | . | . | . | 2 |
| 7058 | . | . | . | . | . | A | C | . | C | . | . | . | . | . | . | A | . | 2 |
| 7067 | . | . | . | . | . | . | . | . | . | . | . | . | . | . | . | A | . | 2 |

**Additional file 4: Table S3.** Mutations in the *MC1R* coding region defining seven haplotypes and their frequencies in Nigerian indigenous pigs

**Note:** 0301 is the European dominant black pigs haplotype; column NIP provides copy number of each haplotype among the samples; dots indicate identity with the previously reported [14] European wild boar haplotype (0101).
